# Analysis of RNA Binding by the Dengue Virus NS5 RNA Capping Enzyme

**DOI:** 10.1371/journal.pone.0025795

**Published:** 2011-10-12

**Authors:** Brittney R. Henderson, Bejan J. Saeedi, Grace Campagnola, Brian J. Geiss

**Affiliations:** 1 Department of Microbiology, Immunology, and Pathology, Colorado State University, Fort Collins, Colorado, United States of America; 2 Department of Biochemistry and Molecular Biology, Colorado State University, Fort Collins, Colorado, United States of America; National Institute of Health, United States of America

## Abstract

Flaviviruses are small, capped positive sense RNA viruses that replicate in the cytoplasm of infected cells. Dengue virus and other related flaviviruses have evolved RNA capping enzymes to form the viral RNA cap structure that protects the viral genome and directs efficient viral polyprotein translation. The N-terminal domain of NS5 possesses the methyltransferase and guanylyltransferase activities necessary for forming mature RNA cap structures. The mechanism for flavivirus guanylyltransferase activity is currently unknown, and how the capping enzyme binds its diphosphorylated RNA substrate is important for deciphering how the flavivirus guanylyltransferase functions. In this report we examine how flavivirus NS5 N-terminal capping enzymes bind to the 5′ end of the viral RNA using a fluorescence polarization-based RNA binding assay. We observed that the K_D_ for RNA binding is approximately 200 nM Dengue, Yellow Fever, and West Nile virus capping enzymes. Removal of one or both of the 5′ phosphates reduces binding affinity, indicating that the terminal phosphates contribute significantly to binding. RNA binding affinity is negatively affected by the presence of GTP or ATP and positively affected by S-adensyl methoninine (SAM). Structural superpositioning of the dengue virus capping enzyme with the Vaccinia virus VP39 protein bound to RNA suggests how the flavivirus capping enzyme may bind RNA, and mutagenesis analysis of residues in the putative RNA binding site demonstrate that several basic residues are critical for RNA binding. Several mutants show differential binding to 5′ di-, mono-, and un-phosphorylated RNAs. The mode of RNA binding appears similar to that found with other methyltransferase enzymes, and a discussion of diphosphorylated RNA binding is presented.

## Introduction

Dengue viruses are members of the *Flaviviridae* family (genus *Flavivirus*), which are small RNA viruses of 10–11 Kb in length with capped non-polyadenylated positive strand genomes. Dengue virus proteins are produced from a single open reading frame via translation of the genomic viral RNA as a single polyprotein that is co-translationally processed into 3 structural proteins (Capsid, prM, and Envelope) and 8 non-structural proteins (NS1, NS2A, NS2B, NS3, NS4A, 2K, NS4B, and NS5). The non-structural proteins are responsible for directing viral genomic RNA replication, including synthesizing negative- and positive-strand RNAs and forming the viral RNA cap structure.

The flavivirus RNA cap is critical for viral polyprotein translation and RNA replication. The RNA cap allows the viral RNA to be efficiently translated by the cellular translational machinery and provides protection for the genome from cellular exonucleases. Flavivirus genomic RNA replication occurs on rough endoplasmic reticulum membranes in membranous compartments away from the cellular capping machinery, requiring the viruses to develop a mechanism for generating an RNA cap structure. Dengue and other flaviviruses have evolved a complete RNA capping machinery to form an RNA cap on the 5′ end of the positive-strand genomic RNA. Cellular RNA cap structures are formed via the action of an RNA triphosphatase (RTPase), guanylyltransferase (GTase), N7-methyltransferase (N7-MTase), and 2′-O methyltransferase (2′O-MTase) [1]. Flavivirus genomic RNA is modified at the 5′ end of positive strand genomic RNA with a cap 1 structure (me^7^-GpppA-me^2^) generated by the virus encoded RTPase (NS3), GTase (NS5), 2′-OMTase (NS5), and Guanine-N7-MTase (NS5) [2], [3], [4], [5], [6], [7], [8], [9]. X-ray crystal structures for each of these viral enzymes have been solved [3], [8], [10], [11] providing a wealth of information about how these enzymes may function. The RTPase resides within the helicase domain of NS3 and appears to utilize the helicase ATP hydrolysis site to remove the γ-phosphate from the 5′ end of the RNA [12]. The NS5 N-terminal capping enzyme domain (dengue virus NS5 AA 1–265) possesses the 2′-O-MTase, Guanine-N7-MTase, and GTase activities and the NS5 C-terminal domain possesses the RNA dependent RNA polymerase [7], [8], [13], [14], [15], [16], [17].

During the GTase reaction, the NS5 N-terminal capping enzyme must bind to the 5′ end of the viral RNA. The GTase reaction uses two substrates, a covalently bound guanosine monophosphate (GMP) and the diphosphorylated 5′ end of the viral genomic RNA, to form the cap 0 structure (5′ GpppAGUAA…). We and others have studied how the capping enzyme binds GTP [8], [11] and the RNA requirements for cap methylation have been explored [16], [17], but there is no empirical evidence for how the protein binds the uncapped diphosphorylated RNA substrate for the GTase reaction. The current location of the RNA binding region has been suggested based on the presence of basic residues and *in silico* molecular dynamics docking of an RNA into the crystal structure of the dengue capping enzyme [18]. A recent structure of the dengue virus type 3 capping enzyme in complex with an octomeric capped RNA demonstrated interactions between the guanosine cap structure and the capping enzyme showed no interactions between the RNA and the capping enzyme putative RNA binding region [19]. This structure may represent the post-capping product, but does not shed light onto how the capping enzyme may bind diphosphorylated RNA during capping. The flavivirus NS5 capping enzyme does not encode a canonical Kx[D/N]G motif or any other known GTase motifs [20], [21], [22], [23]. Since the flavivirus capping enzyme is able to form a guanylated intermediate (a GMP linked to the protein via a phosphoamide bond) and transfer GMP to a diphosphorylated RNA [7], it stands to reason that the capping enzyme must have a non-canonical GTase motif. Understanding how the capping enzyme binds its diphosphorylated RNA substrate is critical for deciphering how this non-canonical GTase functions, but at this point how it binds diphosphorylated RNA is unclear.

In this manuscript we examine the binding of the viral 5′ diphosphorylated RNA substrate to the dengue virus capping enzyme. We developed a fluorescence polarization-based RNA binding assay to monitor the association of a short diphosphorylated RNA corresponding to the conserved 5′ end of the flavivirus genome and determined the RNA binding affinity to the capping enzyme. We assessed the effects of the various ligands used by the capping enzyme on RNA binding affinity, and determined that binding is negatively affected by GTP and ATP and positively affected by SAM. We also performed a structure-directed mutational analysis of the dengue 2 capping enzyme to determine which amino acids may be involved with RNA binding based on the structural similarity of the dengue virus capping enzyme with the Vaccinia virus VP39 methyltransferase protein bound to RNA. We identified several residues that are critical for binding to RNA and report their relative contribution to binding. We have also explored the contribution of the 5′ phosphates to RNA binding and found that the 5′ β- and α- phosphates are critical for diphosphorylated RNA binding to the capping enzyme.

## Materials and Methods

### Expression and purification of flavivirus capping enzyme proteins

Recombinant dengue virus type 2, yellow fever virus, and West Nile virus capping enzymes were previously described [7], [11]. Dengue capping enzyme was produced in BL21 (DE3) pLysS *E. coli* cells (Novagen). Cultures (750 ml) were induced with 400 µM IPTG overnight at 22°C, and the bacterial pellets were collected and stored at −80°C in low imidizole lysis buffer. Frozen pellets were thawed and lysed with a M-110-L Pneumatic microfluidizer (Microfluidics Inc.), and the lysate was clarified by centrifugation at 18 K RPM in a SS-24 rotor and filtered through a 0.22 µM syringe filter. The histidine-tagged protein was purified from clarified lysates using a Hi-Trap Nickel column (GE Healthcare) on an AKTA Purifier FPLC system. The eluted proteins were concentrated using 10 K Amicon Ultra concentrators (Millipore), and buffer exchanged into 400 mM NaCl, 20 mM Tris-Base pH 7.5, 0.02% sodium azide, 20% glycerol, and 5 mM Tris(2-Carboxyethyl) phosphine hydrochloride (TCEP-HCl) on a Superdex 200 gel filtration column (Amersham). Purified proteins were concentrated using 10 K Amicon Ultra concentrators to 100 µM and the concentrations were determined by the absorbance at 280 nm on a NanoDrop 2000 spectrophotometer (Nanodrop, Inc.) using extinction coefficients obtained from the ExPASy web site. Isolated proteins were >98% pure as estimated from SDS-PAGE and Coomassie Blue staining.

### Fluorescence Polarization RNA binding assay

Fluorescence polarization (FP) RNA binding assays were performed with purified wild-type and mutant dengue 2 capping enzymes and 3′-fluoroscein (FAM) labeled 5-base RNAs (5′ ppAGUAA-FAM, 5′ pAGUAA-FAM, 5′ AGUAA-FAM). The RNAs were chemically synthesized (Biosynthesis, Inc), HPLC purified, and verified by mass spectrometry. SAM and SAH were purchased from New England Biolabs. GTP, ATP, GDP, and GMP were purchased from Sigma-Aldrich. All FP experiments were performed in 50 µl volumes in black 384-well microtiter plates. Binding reactions were carried out in final concentrations of 50 mM Tris pH 7.5, 0.1% NP-40, 2 mM DTT, 50 mM NaCl, and 50 nM RNA. Wild-type and mutant dengue capping enzymes were serially diluted in 400 mM NaCl, 50 mM Tris pH 7.5, and 2 mM DTT and added to the RNA mix. The binding reactions were incubated at 28°C for 1 hr, then FP and total fluorescence signals were detected using a Victor 3 V multimode platereader set to 28°C (Perkin Elmer). All FP experiments were performed in triplicate with free RNA and total bound RNA controls. K_D_ values for RNA binding were determined using Kalidagraph (Synergy Software) using an equation based on [24]. In cases where the curves did not reach saturation, we used the average milli-polarization (mP) values for fully bound control samples and force-fitted the curves to those values to estimate K_D_. ppAGUAA-FAM and pAGUAA-FAM had average free and bound mP values of 125 mP and 325 mP, respectively, whereas AGUAA-FAM had average free and bound values of 155 mP and 425 mP.

### Structural Alignment and visualization

Structural alignment of the dengue virus capping enzyme (PDB code: 2P1D) with the Vaccinia VP39 methyltransferase domain (PDB code: 1AV6) was performed with the TopMatch Alignment Server (http://topmatch.services.came.sbg.ac.at) [25], [26]. Structural figures were generated with the PyMOL Molecular Graphics System [27].

## Results

### Development of the fluorescence polarization RNA binding assay

We established a fluorescence polarization-based RNA binding assay to monitor binding of the capping enzyme to the 5′ end of the genomic RNA. The 5′ end of the dengue, yellow fever, and West Nile virus genomic RNA is conserved as an “5′-AG(U/A)” sequence, and we observed that a 5′ ppAG terminated RNA not related to a flavivirus 5′ UTR can be capped by the capping enzyme [7], indicating that only the very 5′ base sequences of the RNA are necessary for the GTase reaction and that RNA structures important for MTase activity are not necessary for the RNA guanyltransfer step [16], [17]. We have used short 5 base AGUAA RNAs with different 5′ ends (5′ppAGUAA, 5′pAGUAA, or 5′AGUAA) and 3′ 6-carboxyfluorescein (FAM) as tools to monitor binding in solution. These RNAs show a polarization of 115–130 mP units in solution when unbound to the capping enzyme, but upon binding polarization signal increases to ∼330 mP. We use this assay in a similar manner to our GTP binding assays described previously [11] and can very accurately and easily monitor RNA binding to the capping enzyme.

**Figure 1:**
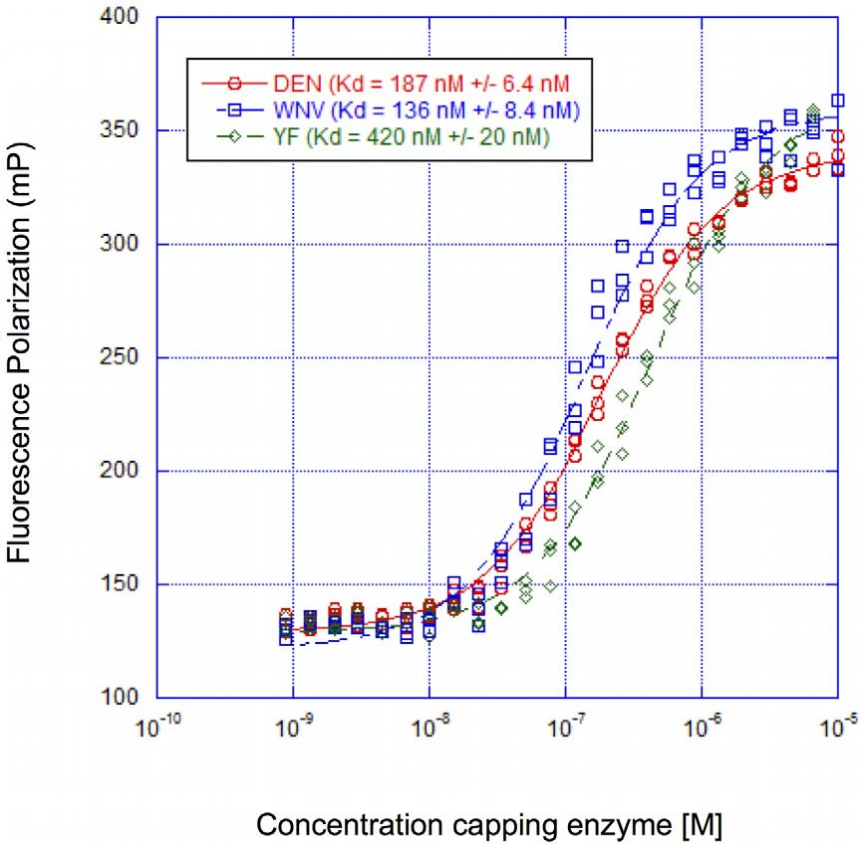
Comparison of dengue, yellow fever, and West Nile virus capping enzyme K_D_ values for ppAGUAA-FAM RNA. 50 nM ppAGUAA-FAM RNA was incubated in increasing concentrations of wild-type capping enzyme for 1 hr, then fluorescence polarization signal was detected. Curve fits and K_D_ values were determined with the KaleidaGraph software package as described in the methods section. n = 3.

We first determined the K_D_ for ppAGUAA RNA binding to dengue 2, yellow fever, and West Nile virus capping enzymes. We obtained similar K_D_ values for ppAGUAA bound to the dengue and West Nile virus proteins (K_D_ = 187±6 nM, and 136±8 nM respectively; Figure 1) and slightly weaker to the yellow fever virus capping enzyme (K_D_ = 420±20 nM, Figure 1). The RNA binds similarly between the three viruses, so we chose to focus on the dengue capping enzyme for the remainder of the project due to its ease of purification and the availability of mutants from our previous work [7], [11].

### Effects of 5′ phosphates on RNA binding

**Figure 2:**
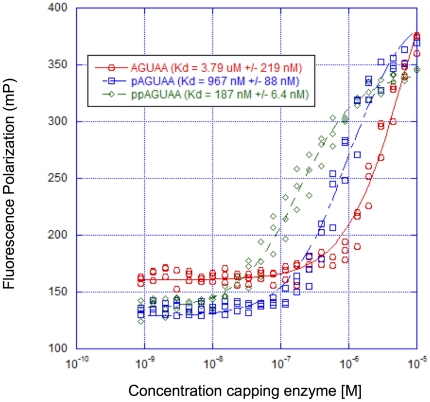
Effects of 5′ RNA phosphates on dengue capping enzyme binding affinity. 50 nM of AGUAA-FAM, pAGUAA-FAM, and ppAGUAA-FAM was incubated with increasing concentrations of wild-type dengue capping enzyme for 1 hr, then fluorescence polarization signal was detected. n = 3.

The presence of two additional phosphates at the 5′ end of the viral RNA likely contribute to the overall binding affinity between the RNA and the capping enzyme. To determine what roles the α- and β-phosphates at the 5′ end of the RNA play in binding, we determined the affinities of ppAGUAA, pAGUAA, and AGUAA RNAs for binding wild-type dengue capping enzyme (187±6 nM, 967±88 nM, and 3.8±0.2 µM respectively) (Figure 2). The Hill slopes of the ppAGUAA and pAGUAA curves are 1.37 and 1.46 as compared to 1.1 for AGUAA, which may indicate very weak positive cooperativity in binding for the 5′ phosphorylated species. However, the Hill slopes for West Nile and yellow fever capping enzymes in Figure 1 were both ∼1.1, so the increased Hill slopes observed in Figure 2 may be dengue specific. These data indicate that the 5′ terminal phosphates contribute significantly to RNA binding, and the α- and β-phosphates both contribute to binding affinity, although β-phosphate appears to contribute more significantly to binding affinity.

### Structural superposition of the Vaccinia Virus VP39 with the yellow fever capping enzyme

**Figure 3:**
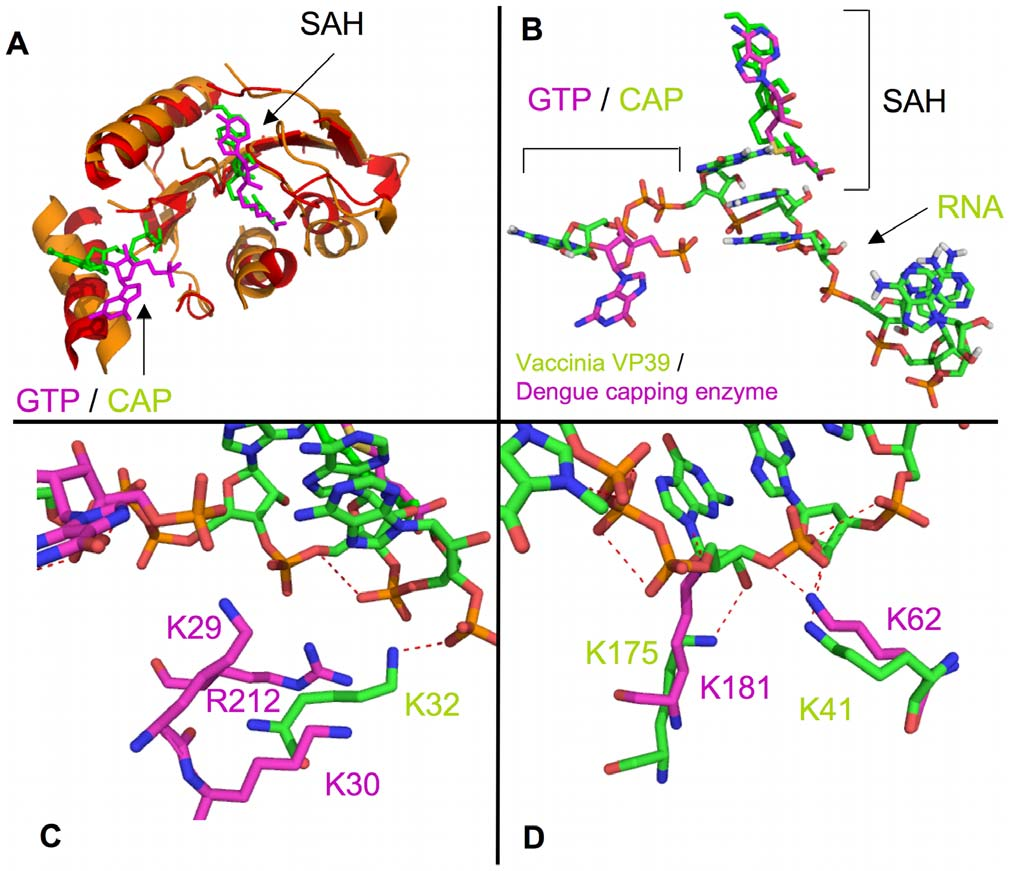
Structural superposition of the Vaccinia virus VP39 with the dengue virus capping enzyme. **A**) Global overlap of 1AV6 (VP39) and 2P1D (dengue virus capping enzyme). Superposition was performed using the TopMatch webserver, and figures were generated in PyMol. Red/orange indicate structural overlap between 1AV6 and 2P1D. Non-overlapping regions are not shown. Bound GTP/Cap and SAH are shown. RNA has been removed for clarity. **B**) Overlay of bound ligands from 1AV6 (green) and 2P1D (magenta). **C**) Overlap of 1AV6 residue K32 (interacting with RNA phosphate #6) to 2P1D residues K29, K30, and R212. **D**) Overlap of 1AV6 residues K41 (interacting with RNA phosphate #4) and K175 (interacting with RNA ribose #1 hydroxyl) to 2P1D K62 and K181, respectively.

The flavivirus capping enzyme does not have structural homology to known GTases but does have significant structural homology to methyltransferase enzymes. We performed a TopMatch structural alignment with the Vaccinia virus VP39 methyltransferase protein (PDB code: 1AV6) [28] which was crystallized with a bound capped RNA, and the dengue virus capping enzyme in complex with GTP (PDB code: 2P1D) (Figure 3). The alignment showed strong structural homology in the methyltransferase section of the capping enzyme (Figure 3A), but more interestingly the bound cap/GTP and SAH ligands were in very similar positions in both structures, and the RNA bound in 1AV6 is in close proximity to the basic patch of residues that has been postulated to be the RNA binding site (Figure 3B). Residues K32, K41, and K175 in the VP39 structure (1AV6) appear to interact with the phosphates of the RNA. Dengue virus capping enzyme residues K62, K181, and R212/K29/K30 appear to structurally overlap with VP39 residues K41, K175, and K32, respectively. The R212/K29/R30 cluster is slightly farther away from the RNA than VP39 K32 (Figure 3C), but would strongly interact with the RNA if it bent towards the residues. K181 (dengue) appears to structurally clash with the RNA ribose group in the 1AV6 structure, whereas K175 (VP39) interacts with the ribose (Figure 3D). This may indicate that the RNA would need to be pushed away from its position in the VP39 structure to accommodate binding to K181.

### Mutational Analysis of dengue virus capping enzyme RNA binding

Based on the structural alignment of VP39 and the dengue virus capping enzyme, we performed a mutagenesis analysis of the dengue capping enzyme to evaluate individual residue contributions to RNA binding. Protein∶RNA interactions commonly occur between basic residues (Arg and Lys) interacting with phosphates within and at the end of the RNA. The region between the GTP and SAM binding site on the capping enzyme is rich with basic residues, and has been hypothesized to be the RNA binding site. To examine the contribution of residues to RNA binding, we individually mutated conserved and semi-conserved (eg. K/R) residues on the GTP/SAM binding face of the capping enzyme and determined how each mutation affected binding affinity di-, mono-, and unphosphorylated RNAs (Table 1).

**Table 1:** Binding affinities of mutant dengue capping enzyme proteins for different diphosphorylated RNA species and GTP.

	AGUAA		pAGUAA		ppAGUAA		GTP	
Mutant	Average K_D_	SD	Average K_D_	SD	Average K_D_	SD	Average K_D_	SD
WT	3.7 µM	217 nM	967 nM	88 nM	187 nM	6 nM	77 nM	4 nM
K22A	3.9 µM	470 nM	862 nM	201 nM	689 nM	450 nM	120 nM	24 nM
F25A	6.0 µM	687 nM	1.8 µM	440 nM	1.4 µM	501 nM	4.6 µM	495 nM
K29A	4.1 µM	1.0 µM	1.4 µM	75 nM	652 nM	59 nM	609 nM	154 nM
K30A	18.5 µM	4.7 µM	1.8 µM	243 nM	4.0 µM	778 nM	133 nM	41 nM
E35A	3.4 µM	813 nM	524 nM	69 nM	247 nM	30 nM	67 nM	15 nM
R57A	2.1 µM	462 nM	735 nM	137 nM	2.66 µM	775 nM	78 nM	17 nM
G58A	2.9 µM	337 nM	687 nM	71 nM	641 nM	54 nM	85 nM	36 nM
K62A	5.5 µM	299 nM	1.3 µM	649 nM	305 nM	122 nM	271 nM	59 nM
K181A	8.5 µM	2.0 µM	2.75 µM	742 nM	4.6 µM	1.9 µM	359 nM	32 nM
R212A	7.9 µM	845 nM	1.6 µM	79 nM	1.0 µM	222 nM	193 nM	31 nM

50 nM of the indicated AGUAA-FAM, pAGUAA-FAM, ppAGUAA-FAM, or 10 nM GTP-Bodipy were incubated with increasing concentrations of the indicated protein for 1 hr at 28°C then fluorescence polarization signal was detected. K_D_ and standard deviation values are reported for each. n = 3.

We observed that mutation of residues F25, K30, R57, K181, and R212 reduced the binding affinity to the greatest extent of all residues tested (≤5-fold reduction of K_D_ (Table 2)). The remaining mutations had little effect on RNA binding affinity. K62A in the proposed RNA bind site did not affect RNA binding with any RNA species, indicating that it is not involved in binding RNA. K30A appeared to have a strong effect on binding with ppAGUAA and AGUAA, indicating that it interacts with a phosphate present in both RNA species, most likely the phosphate between the A and G bases of the AGUAA RNA. F25A was initially added as a control because it interacts with the guanosine cap that is not present on the RNAs in this study. However, we observed that the mutant had significantly reduced binding to ppAGUAA RNA but no significant effect on pAGUAA and AGUAA binding. The phenylalanine group likely does not interact directly with the RNA, but a possible explanation is that mutation to alanine alters the position of helix A2 and moves K30 out of the optimal position to keep the 5′ diphosphate in line with other binding residues. R57A appeared to have its greatest effect when binding to ppAGUAA but less effect with pAGUAA and AGUAA, suggesting that R57 may interact predominately with the β-phosphate of the diphosphorylated RNA. K181A severely reduced ppAGUAA binding but had greatly reduced effects on pAGUAA and AGUAA binding, suggesting that K181 strongly interacts with the β-phosphate. R212A showed significant effects with binding to ppAGUAA and mild effects on pAGUAA binding (2.8 fold), suggesting that R212 interacts primarily with the β-phosphate but may weakly interact with the α-phosphate. In summary, based on these experiments R57, K181, and R212 likely interact with the β-phosphate, R212 may interact with the α- or β-phosphate, and K30 may interact with the phosphate between the A and G bases.

**Table 2:** Comparison of RNA and GTP binding affinities.

Mutant	Ratio Mutant/WT AGUAA K_D_	Ratio Mutant/WT pAGUAA K_D_	Ratio Mutant/WT ppAGUAA K_D_	Ratio Mutant/WT GTP K_D_
WT	1.0	1.0	1.0	1.0
K22A	1.1	0.9	3.7	1.6
F25A	1.6	1.9	7.3	60.3
K29A	1.1	1.4	3.5	7.9
K30A	5.0	1.9	21.2	1.7
E35A	0.9	0.5	1.3	0.9
R57A	0.6	0.8	14.2	1.0
G58A	0.8	0.7	3.4	1.1
K62A	1.5	1.3	1.6	3.5
K181A	2.3	2.8	24.5	4.7
R212A	2.1	1.7	5.5	2.5

Fold change was determined for RNA binding by comparing each mutant K_D_ value to wild-type (WT) ligand binding value from Table 1.

### Effects of GTP and SAM on RNA binding affinity

**Figure 4:**
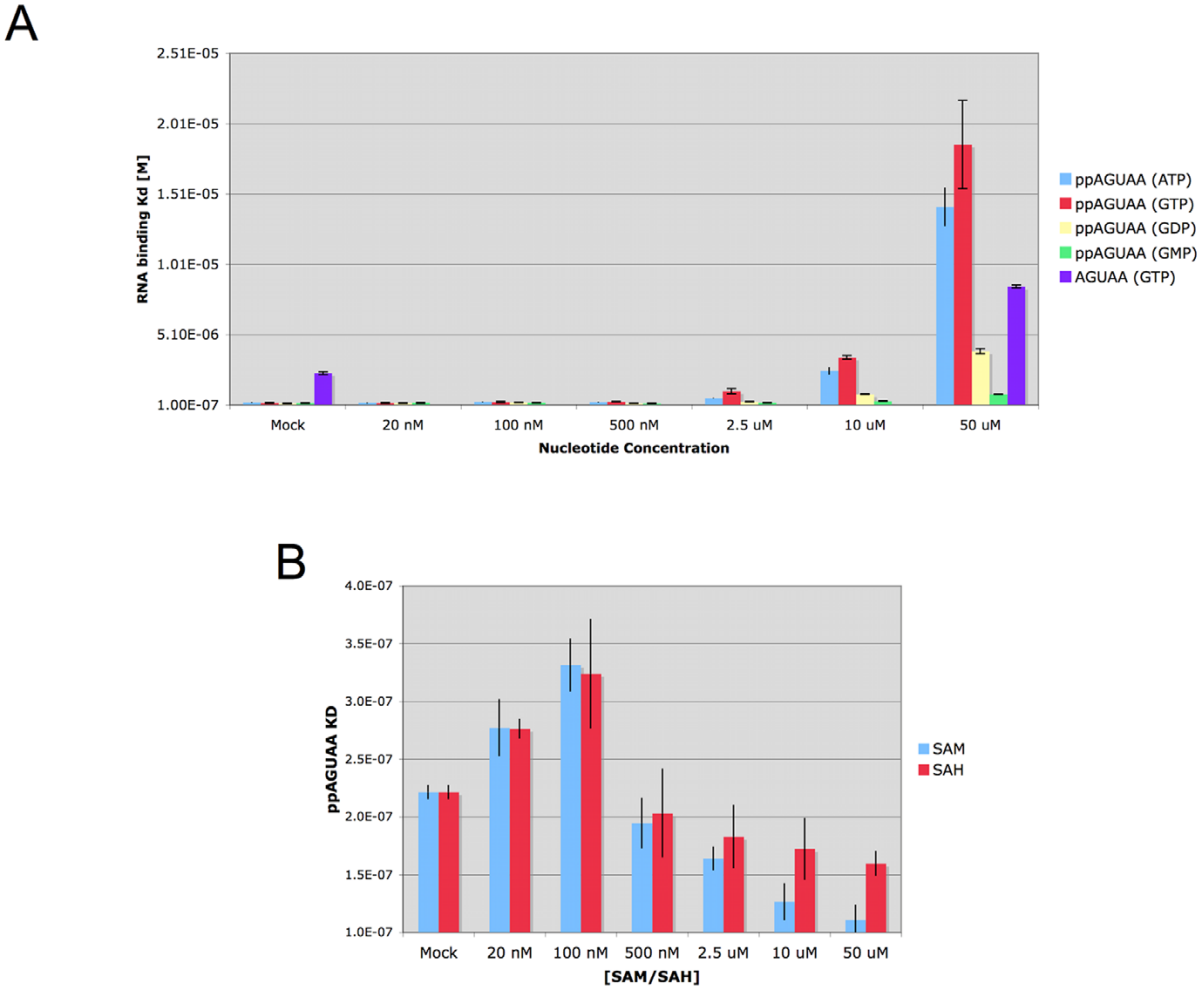
Effects of capping enzyme ligands on RNA binding. **A**) Effect of purine nucleotides on ppAGUAA-FAM and AGUAA-FAM RNA binding. K_D_ values for ppAGUAA binding to wild-type dengue capping enzyme were determined in the presence of increasing concentrations of the indicated nucleotide. AGUAA binding was determined only in the presence of 50 µM GTP or Mock. **B**) Effect of SAM and SAH on ppAGUAA-FAM RNA binding affinity. n = 3.

Diphosphorylated RNA is one of three ligands involved in the guanyltransferase and methyltransferase reactions, the other two being GTP and SAM. We assessed the effects of GTP and SAM on RNA binding to wild-type dengue capping enzyme to determine if either could positively or negatively affected binding affinity. We first determined the K_D_ of ppAGUAA in presence of increasing amounts of GTP, GDP, GMP, and ATP (Figure 4A). We observed that high concentrations of GTP and ATP (50 µM) significantly weakened ppAGUAA binding (K_D_ = ∼18 µM), whereas GDP had a moderate affect on ppAGUAA binding binding (K_D_ = ∼3 µM) and GMP had a very minor effect on ppAGUAA binding (K_D_ = ∼800 nM). The effect of GTP and ATP on ppAGUAA RNA binding (an 80 fold reduction in binding) as compared to GDP and GMP levels suggest that the γ- and β-phosphates on the nucleotides compete with the diphosphate on the RNA for binding. To further examine if the GTP β- and γ- phosphates compete with diphosphorylated RNA binding, we determined the affinity of AGUAA RNA in the presence of 50 µM GTP. We observed that AGUAA binding was weakened 2.4 fold in the presence of 50 µM GTP (K_D_ (Mock) = 3.5 µM, K_D_ (50 µM GTP) = 8.4 µM), indicating that GTP had only minor effects on AGUAA binding. These results suggest that the GTP phosphates interfere with the diphosphate on the ppAGUAA RNA during binding.

To examine the effect of SAM and the post-methylation product SAH, we determined the K_D_ of ppAGUAA binding to the dengue capping enzyme in the presence of increasing amounts of SAM or SAH (Figure 4B). At low concentrations of SAM and SAH we observed no effect of either SAM or SAH on ppAGUAA binding affinity (K_D_ = ∼200 nM), but we did observe a slight increasing in ppAGUAA RNA affinity in the presence of increasing concentrations of both SAM and SAH. A slightly stronger effect on RNA binding affinity was evident with SAM than SAH, suggesting that SAM was able to stabilize RNA binding slightly better than SAH.

### Mapping diphosphorylated RNA binding to the flavivirus capping enzyme

**Figure 5:**
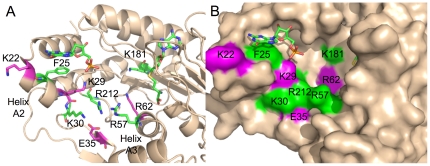
RNA binding residues on the dengue capping enzyme. All residues that were tested in this study were mapped on the dengue virus capping enzyme structure (2P1D) bound to GTP [11]. **A**) Residues that showed greater than 5-fold reduction in RNA binding affinity against AGUAA, pAGUAA, or ppAGUAA are colored in green. Residues that showed less than 5-fold reduction in binding affinity against AGUAA, pAGUAA, or ppAGUAA are colored in magenta. Bound GTP and SAH are shown. **B**) Surface representation of 2P1D with RNA binding residues colored green and non-binding residues colored magenta.

Based on our biochemical data, we mapped residues that significantly interacted with RNAs on RNA binding residues on the dengue virus capping enzyme in complex with GTP (PDB code: 2P1D). Figure 5 shows residues that were tested in this manuscript, and color codes the effects of each residue on RNA binding. Residues K22, K29, E35, and R62 had minimal effects on RNA binding (magenta), whereas residues F25, K30, R57, K181, and R212 (green) had significant effects on binding affinities (reduction greater than 5-fold). A clear clustering around the base of helix A2 is apparent, and suggests that the diphosphorylated RNA may enter the capping enzyme to be capped through the groove region between helices A2 and A3.

## Discussion

In this study we performed a detailed characterization of the RNA binding characteristics of the dengue 2 capping enzyme. We present data demonstrating that dengue, yellow fever, and West Nile virus capping enzyme proteins bind 5′ diphosphorylated end of the viral RNA with similar affinity, although yellow fever virus capping enzyme bound RNA with 2-fold weaker affinity than dengue or West Nile virus capping enzymes. The yellow fever virus capping enzyme has an arginine at position 30 whereas the dengue and West Nile virus capping enzymes have a lysine at position 30, which may explain the small difference in affinity between the viruses (Figure 1). Based on our biochemical and mutagenesis data, we present a preliminary model for capping enzyme binding to the 5′ diphosphorylated end of the viral genomic RNA strand (Figure 5). The mechanism of action for the guanylyltransferase and methyltransferase activities of the flavivirus NS5 capping enzyme have been the focus of intensive study since the dengue capping enzyme structure was solved in 2002 [8], [13], [16], [17]. Most of the effort has been focused on understanding the methyltransferase function. Because how the GTase activity within the capping enzyme functions is unknown, understanding how the capping enzyme binds one of its GTase substrates will help clarify how the enzyme works.

The overlap of the Vaccinia VP39 protein with the dengue capping enzyme is very strong in the methyltransferase region of the capping enzyme as had been previously noted, and superposition and alignment of residues involved in RNA cap binding have been described [8]. The location of the SAH in both structures is almost identical, and the GTP in the dengue virus capping enzyme is in close proximity to the cap structure in VP39. An obvious difference between the two structures is that the GTP/Cap structures are flipped in respect to each other, indicating differing modes of guanine recognition. The VP39 cap and the dengue virus capping enzyme GTP are shifted approximately 5 Å from each other, indicating that a capped RNA bound to the dengue virus capping enzyme would also be shifted ∼5 Å from where the RNA in the VP39 structure is situated.

Mutation of K62 in the dengue capping enzyme did not affect RNA binding, whereas the homologous lysine residue in the Vaccinia virus VP39 did interact with a phosphate (Figure 3D). K181 mutation strongly affected binding, suggesting that the superposition was partially correct. We observed a steric clash between K181 and the superimposed VP39 RNA, suggesting that the mode of K181 (dengue) interaction with RNA may be somewhat different than K175 (VP39) binding to the ribose of the first RNA nucleotide. The dramatic effect of mutating K30 on RNA binding suggests that the K30 strongly interacts with the RNA. VP39 K32, which partially overlaps with the K29/K30/R212 cluster, interacts with a phosphate, but K30 and R212 are situated about 5 Å away from the superimposed RNA. The lack of effect by mutating K62 and the strong effect by mutating K30 and R212 (to a lesser extent) suggest that the RNA is pulled back toward K30/R212 and away from K62 in the dengue capping enzyme structure. Supporting these observations is the effect seen on diphosphorylated RNA binding to the F25A mutant. This mutation severely affects GTP binding, but also appears to significantly affect RNA binding. Removing a hydrophobic phenylalanine likely perturbs the stability of the helix A2 (K30 is at the base of helix A2 (Figure 5)) and potentially reduces K30 binding the RNA by reducing its stability or moving it out of optimal binding position. Combining the differences in the locations of the cap and GTP with the effects on binding seen with K30 and R212 but not K62 suggests that the capped RNA would wrap tightly around helix A2 in the capping enzyme and have little to no interaction with the SAM binding face of the capping enzyme. This does not preclude the RNA ribose hydroxyl groups from being in an appropriate position to undergo 2′-O methylation, but additional structural studies would need to be performed to determine their positions.

Addition of GTP and ATP to the ppAGUAA-FAM binding experiments showed that the phosphates on the nucleotides are able to compete with diphosphorylated RNA binding, and that reducing the number of phosphates on the nucleoside reduces the observed competitive effect. We also observed that high concentrations of GTP had only minor effects on the binding of an unphosphorylated AGUAA RNA, suggesting that GTP predominately interferes with diphosphate binding. The position of GTP binding to the capping enzyme is well known [8], [11], and GTP phosphates interact with several residues that are also involved in RNA interaction based on this study. R212 and K181 bind to GTP phosphates as well as to RNA (Table 1 and [11]), suggesting that the residues could interact with GTP or RNA at different times during capping. Since GTP was able to displace diphosphorylated RNA from the capping enzyme, it is likely that the capping enzyme would first bind GTP and form a guanylated protein intermediate prior to interacting with the diphosphorylated RNA to form the cap. This could inform the order of substrate binding during the GTase reaction, which would help with the development of a catalytic mechanism for the GTase. The small increase in ppAGUAA-FAM affinity that was observed in the presence of SAM suggests either that SAM stabilizes the interaction between the RNA and the capping enzyme or that SAM transfers a methyl group to the RNA that increases the affinity of the RNA for the capping enzyme. The effect observed with SAM may have a component of each situation, as SAH (which cannot methylate the RNA) show a weak stabilizing effect on RNA binding. We are currently testing if the diphosphorylated RNA is methylated in the presence of SAM by mass spectrometry. If we determine that the diphosphorylated RNA substrate is methylated in the presence of SAM, it would suggest that the genomic RNA may be methylated at the 2′-O position prior to RNA capping, providing important information about the order of cap formation.

These studies provide a solid foundation for further exploration into how the flavivirus capping enzymes bind to the 5′ diphosphorylated end of the viral genomic RNA during RNA capping. It provides a starting point for further investigations into to determinants of RNA binding, such as further clarifying which sidechains interact with which phosphate, determining the role of ribose hydroxyls in binding, and the specificity for guanine versus adenine in the first nucleotide position during capping [7]. Additional studies will help clarify these questions. Testing ppAGUAA-FAM RNAs with specific methylphosphanate substitutions in combination with capping enzyme mutants would help to map out specific amino acid∶phosphate interactions [29]. Substituting ribose groups with 2′ deoxy ribose at specific positions in the RNA would allow for an examination of hydroxyl interactions with specific amino acids. Substitution of each base in the AGUAA RNA with analogs (eg. ppIGUAA-FAM, where I = inosine) would be critical for understanding the correlates of adenine vs. guanine specificity for capping the RNA [7]. With these data, molecular docking experiments could be performed that take into account the various distance restraints to build a biochemically-derived model of RNA binding. Our increased understanding of how the flavivirus capping enzyme binds to the substrates it uses to cap the genome will aid in our understanding of how this non-canonical capping enzyme functions, and will be valuable for the development of rationally designed capping enzyme specific drugs.
